# Superhydrophobic Dressing for Singlet Oxygen Delivery in Antimicrobial Photodynamic Therapy against Multidrug-Resistant Bacterial Biofilms

**DOI:** 10.1021/acsabm.4c00733

**Published:** 2024-08-21

**Authors:** Fernanda V. Cabral, QianFeng Xu, Alexander Greer, Alan M. Lyons, Tayyaba Hasan

**Affiliations:** † Wellman Center for Photomedicine, Massachusetts General Hospital and Harvard Medical School, 40 Blossom Street, Boston, Massachusetts 02114, United States; ‡ SingletO2 Therapeutics LLC, VentureLink, Room 524B, 211 Warren Street, Newark, New Jersey 07103, United States; § Ph.D. Program in Chemistry, The Graduate Center of the City University of New York, 365 Fifth Avenue, New York, New York 10016, United States; ∥ Department of Chemistry, Brooklyn College, City University of New York, Brooklyn, New York 11210, United States; ⊥ Department of Chemistry, College of Staten Island, City University of New York, Staten Island, New York 10314, United States; # Division of Health Sciences and Technology, Harvard University and Massachusetts Institute of Technology, Cambridge, Massachusetts 02139, United States

**Keywords:** drug-resistant biofilms, verteporfin, *Pseudomonas aeruginosa*, *Staphylococcus aureus*, wound healing

## Abstract

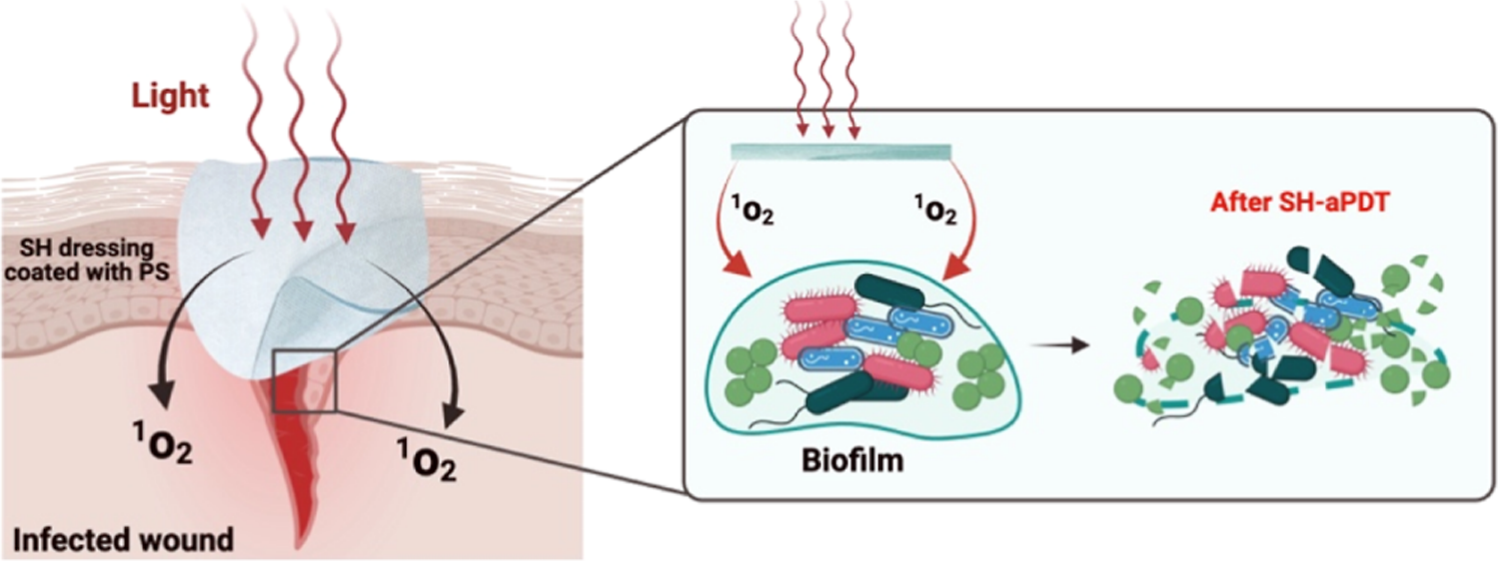

The rise of antimicrobial resistance poses a critical public health threat worldwide. While antimicrobial photodynamic therapy (aPDT) has demonstrated efficacy against multidrug-resistant (MDR) bacteria, its effectiveness can be limited by several factors, including the delivery of the photosensitizer (PS) to the site of interest and the development of bacterial resistance to PS uptake. There is a need for alternative methods, one of which is superhydrophobic antimicrobial photodynamic therapy (SH-aPDT), which we report here. SH-aPDT is a technique that isolates the PS on a superhydrophobic (SH) membrane, generating airborne singlet oxygen (^1^O_2_) that can diffuse up to 1 mm away from the membrane. In this study, we developed a SH polydimethylsiloxane dressing coated with PS verteporfin. These dressings contain air channels called a plastron for supplying oxygen for aPDT and are designed so that there is no direct contact of the PS with the tissue. Our investigation focuses on the efficacy of SH-aPDT on biofilms formed by drug-sensitive and MDR strains of Gram-positive (*Staphylococcus aureus* and *S. aureus* methicillin-resistant) and Gram-negative bacteria (*Pseudomonas aeruginosa* and *P. aeruginosa* carbapenem-resistant). SH-aPDT reduces bacterial biofilms by approximately 3 log with a concomitant decrease in their metabolism as measured by MTT. Additionally, the treatment disrupted extracellular polymeric substances, leading to a decrease in biomass and biofilm thickness. This innovative SH-aPDT approach holds great potential for combating antimicrobial resistance, offering an effective strategy to address the challenges posed by drug-resistant wound infections.

## Introduction

Antimicrobial resistance poses a significant global health threat, impacting not only individual patients but also having broader consequences for public health.^1^ It has led to substantial economic impacts as treating resistant infections becomes more challenging and expensive, resulting in increased healthcare costs and elevated mortality rates.^1,2^

The emergence of multidrug-resistant (MDR) bacteria has gained significant attention in recent years. It has been attributed to factors such as misuse/overuse of antibiotics, inappropriate prescribing, and the lack of development of new drugs.^1−3^ These factors enable the bacteria to adapt and develop resistance mechanisms, including the overexpression of efflux pumps and reduction in drug internalization.^3^

Moreover, bacteria can form biofilms, presenting an additional challenge to treatment.^4^ Biofilms, existing in an attached and sessile state, differ from planktonic cells due to their numerous upregulated genes, degradation enzymes, and the presence of extracellular polymeric substances (EPS) that contribute to their protection, proliferation, and dissemination.^4,5^ In the context of wound infections, biofilms play a major role and can delay the healing process.^5^ Pathogenic biofilms pose a threat as they can be resistant to antimicrobial drugs and more tolerant to immune responses, hindering wound healing and leading to chronic inflammation, sepsis, and death.^4,5^

Since antibiotics are the primary treatment for bacterial-related infections, there is an urgent need for alternative strategies to tackle the problem of drug resistance. Antimicrobial photodynamic therapy (aPDT) has emerged as an attractive light-based technology to treat localized infections, as it can kill a wide range of pathogens through oxidative stress.^6^ aPDT produces reactive oxygen species, including the highly reactive singlet oxygen (^1^O_2_), which is a potent oxidant within microorganisms.^6^ However, ^1^O_2_ has a very short 3.5 μs lifetime and diffuses short distances of ∼100–200 nm in H_2_O, making its interaction with pathogens dependent on photosensitizer (PS) localization and accumulation.^6−12^

Moreover, bacteria can develop resistance to PSs via mechanisms such as efflux pumps.^13,14^ aPDT may also be limited by the conditions of the infected tissue as the PS is likely to bind the biological components present in inflammatory exudates rather than only bacterial membranes, thus decreasing the availability of PS to bind and kill pathogens.^15^

**Figure 1:**
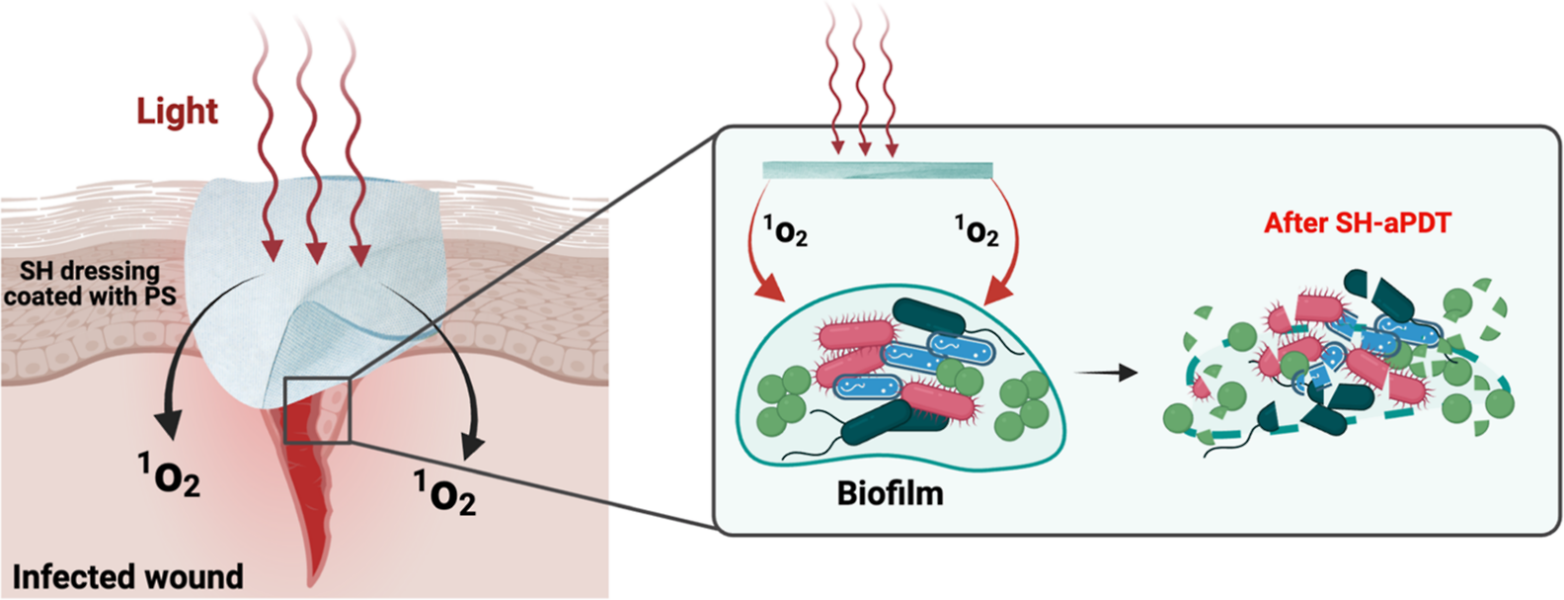
Schematic of an SH-aPDT dressing used to disinfect a wound by generating airborne ^1^O_2_ when illuminated with visible light of the appropriate wavelength. The airborne ^1^O_2_ diffuses from the bandage to the wound surface, where it reacts with the biofilm to kill both Gram-positive and Gram-negative bacteria as well as destroy the surrounding EPS.

To address these limitations, we developed a new aPDT technique that delivers airborne ^1^O_2_ to the infection site while retaining the PS on superhydrophobic (SH) surfaces. This superhydrophobic antimicrobial photodynamic therapy (SH-aPDT) method was used to kill various bacterial biofilms with promising results.^16−18^ These surfaces can work as dressings and contain air channels that supply sufficient oxygen to react with the PS upon light illumination, as shown schematically in Figure 1.^9,16−18^ The airborne ^1^O_2_ diffuses approximately 1 mm in the air without the PS directly contacting the tissue, thus offering a new strategy to overcome some of the aPDT limitations.^17−19^

We recently demonstrated the potential of this innovative treatment in vivo. By using Wistar rat models with periodontitis, it was observed that SH-aPDT not only significantly decreased *Pseudomonas gingivalis* biofilms but also reduced inflammation, resulting in an increase in fibroblast cells, thus allowing complete tissue healing.^17^ Further investigation is required to explore the effectiveness of SH-aPDT on the MDR biofilms.

In this work, we developed freestanding SH polydimethylsiloxane (PDMS) films coated with PS verteporfin (VP) at loadings of 12, 60, and 300 μg/cm^2^. We note that VP is a constituent of the FDA-approved PS emulsion visudyne.^9^ Our investigation focuses on the efficacy of SH-aPDT on biofilms formed by drug-sensitive and MDR strains of Gram-negative (*Pseudomonas aeruginosa* PAO1, *P. aeruginosa* carbapenem-resistant, CRPA) and Gram-positive bacteria (*Staphylococcus aureus* ATCC and *S. aureusmethicillin*-*resistant*, MRSA).

SH-aPDT was performed using a red laser emitting at a peak of 690 ± 3 nm to match the absorbance of the PS. To evaluate the antimicrobial potential of SH-aPDT, we used the colony-forming unit (CFU) method to estimate the number of viable cells and confocal microscopy using LIVE/DEAD staining. We also aimed to investigate the efficacy of SH-aPDT on biofilm metabolic activity using an MTT assay. Biofilm biomass was evaluated using the crystal violet (CV) method and confocal microscopy for EPS staining. Additionally, a quenching study was conducted to demonstrate that singlet oxygen is the initial species involved in bacterial killing, followed by oxygen radicals from bioperoxide decomposition based on studies with HPF.

## Methods

### Bacteria Culture and Biofilm Formation

The bacteria species *P. aeruginosa* (PAO1), *P. aeruginosa* carbapenem-resistant (CRPA), and *S. aureus* (ATCC25923 and MRSA-USA300) were used. Biofilms were grown and cultured in brain heart infusion broth (BHI) or BHI supplemented with 15 g/L agar at 37 °C. Cell densities of the bacterial suspensions were adjusted to 1 × 10^6^ CFU/mL and seeded in 96-well plates. After 24 h of incubation, the media were refreshed, and biofilms were incubated for another 24 h, thus resulting in 48 h of incubation time. Then, biofilms were gently washed twice with PBS, and SH membranes were placed on the top of the biofilms to be further irradiated.

### Susceptibility Tests

Antimicrobial susceptibility tests were conducted to assess the minimum inhibitory concentration (MIC) values of various clinically relevant antibiotics. The MIC values were determined using the broth microdilution method, according to the guidelines provided by the Clinical and Laboratory Standards Institute.^20^

### Efficacy of SH-aPDT against Bacterial Biofilms

The laser emitting at a peak of 690 ± 3 nm was set to deliver 3 different incident fluences (100, 200, and 300 J/cm^2^) at an incident irradiance of 160 mW/cm^2^. These fluences resulted in an illumination time of 625, 1250, and 1875 s, respectively. Five control groups were evaluated: nontreated control (here defined as NT), SH PDMS surfaces without PS and without light (SH+PS–L−), SH PDMS surfaces without PS but with light at a transmitted fluence (TF) of 155.7 J/cm^2^ (SH+PS–L+), SH surfaces coated with three different VP loading levels (12, 60, and 300 μg/cm^2^) without light exposure (SH+PS+L−), and light only at 300 J/cm^2^. Because of the PS coating, PDMS dressings can diminish the amount of light transmitted through the SH surface. Different concentrations resulted in different TFs. Therefore, the TF was calculated and described in Table 2. The experimental groups and exposure time corresponding to each light dose are described in Tables 1 and 2, respectively.

**Table 1:** Experimental Control and SH-aPDT Groups

groups	comment
NT	no treatment
SH–PS–L+	light at fluence 300 J/cm^2^
SH+PS–L–	SH PDMS without PS and no light
SH+PS–L+	SH PDMS without PS but with light
SH+PS+L–	SH PDMS with PS (12, 60, 300 VP) without light
SH+PS+L+	SH PDMS with PS (12, 60, 300 VP) and with light at different transmitted fluences (Table 2)

**Table 2:** Transmitted Irradiance and Fluence through SH Dressings Coated with Different Concentrations of VP

SH dressing type	verteporfin concentration (μg/cm^2^)	transmitted irradiance (mW/cm^2^)	incident fluence (J/cm^2^)	transmitted fluence (J/cm^2^)
SH+PS–L+	none	83	100	51.9
			200	103.7
			300	155.7
SH+PS+L+	12	78.5	100	49.0
			200	98.1
			300	147.2
SH+PS+L+	60	70.4	100	44.0
			200	88.0
			300	132.0
SH+PS+L+	300	53.1	100	33.2
			200	66.4
			300	99.6

After SH-aPDT, wells were scraped with a sterile pipet tip, resuspended in PBS, and sonicated for 5 min. Then, 10-fold serial dilutions were conducted for each group for colony counting, determined as colony-forming units per mL (CFU/mL).

### MTT Assay

To evaluate the bacterial metabolic activity, an MTT [3-(4,5-dimethylthiazol-2-yl)-2,5-dyphenyltetrazolium bromide] assay was performed. MTT is a common method used to evaluate the cell viability and metabolic activity in various biological systems, including biofilms. The reduction of MTT in bacterial specimens involves various metabolic pathways and enzymes. For this, biofilms of *P. aeruginosa* PAO1, CRPA, *S. aureus* ATCC, and MRSA were exposed to SH-aPDT at different VP concentrations and light doses, as described above. After treatment, biofilms were carefully washed with PBS, and 0.5 mg/mL MTT was added to each well for 4 h at 37 °C. Then, 100 μL of DMSO was added for 30 min, and absorbance was measured with a plate reader at 570 nm.

### Confocal Microscopy

Confocal microscopy was used to assess the biofilm viability and thickness. Biofilms of all 4 strains were treated with SH-aPDT at 132 J/cm^2^ and 60 μg/cm^2^ VP. Then, biofilms were washed twice with PBS to remove nonadherent cells and 200 μL of distilled water containing LIVE/DEAD dye BacLight bacterial viability assay (Molecular Probes, Inc.) containing SYTO9, and propidium iodide (PI) was added to the wells. After 15 min, specimens were rinsed with PBS and evaluated under confocal microscopy (Olympus FV1000) at wavelengths of 488 nm excitation/500 nm emission for SYTO9 and 535 nm excitation/635 nm emission for PI. Biofilm thickness was assessed by performing 3D confocal imaging using 1 μm stacks.

To analyze the structure of the EPS matrix, biofilms were exposed to the same conditions as described above. Then, they were washed twice with PBS to remove nonadherent cells, and 200 μL of distilled water containing FilmTracer SYPRO Ruby biofilm matrix stain (Invitrogen, Inc.) was added to the wells. After 30 min, specimens were rinsed with PBS and evaluated under confocal microscopy (Olympus FV1000) at 450 nm excitation/610 ± 30 nm emission.

### CV Staining

Biofilm biomass was evaluated by the addition of 100 μL of CV at 0.5% to each well immediately after SH-aPDT. The plate was incubated for 10 min at 25 °C. CV was removed, and the sample was washed three times with PBS. Then 100 μL of 33% acetic acid was added per well to dissolve the biofilm. Absorbance was measured at 595 nm.

### Singlet Oxygen Quenching Studies

For the quenching studies, 48 h biofilms were grown in BHI at 37 °C. Prior to irradiation, the media were replaced with media containing sodium azide, selecting a concentration of 2.0 mM,^21,22^ followed by placement of the SH membrane on top of the biofilm.^21^ Thereafter, they were exposed to the best SH-aPDT conditions (132 J/cm^2^ and 60 μg/cm^2^ VP). Wells were then scraped, resuspended in PBS, and sonicated for 5 min. Then, 10-fold serial dilutions were performed for colony counting (CFU/mL).

### Assessing Bioperoxide Decomposition by HPF

Biofilms were grown and washed as previously described to seek evidence for downstream oxygen radicals formed by the decomposition of the initially photogenerated bioperoxides after airborne ^1^O_2_ exposure. Here, the fluorescence dye 2-[6-(4′-hydroxy)phenoxy-3*H*-xanthen-3-on-9-yl]benzoic acid (HPF) (Invitrogen) (10 μM) was used by adding to the wells containing the biofilms and incubating at 37 °C for 30 min. Biofilm specimens were then illuminated with a red laser at three SH-aPDT (4.2, 44, and 88 J/cm^2^) conditions along with controls without SH surfaces. The incident fluence for the lowest dose was 20 J/cm^2^, which corresponds to a TF of 4.2 J/cm^2^. Evidence for radicals was detected by using a plate reader.^23^ The excitation/emission wavelengths were 490 nm/515 nm, respectively.^8^

### Statistical Analysis

Statistical analysis was evaluated by using GraphPad Prism 10 software by one-way analysis of variance (ANOVA), followed by the Tukey post-test for CFU counting, biofilm thickness, quenching studies, and peroxide radicals. For metabolic activity and biofilm biomass, statistical analysis was assessed by two-way ANOVA, followed by the Tukey post-test. Differences were considered statistically significant when *p* < 0.05.

## Results

### Antibiotic Susceptibility of Bacterial Strains

Table 3 shows the MIC of various antibiotics against different bacterial strains, highlighting their susceptibility or resistance.

**Table 3:** MIC of Various Antibiotics for All Four Bacterial Strains^a^

antibiotic	MIC in μg/mL			
	S. aureus ATTC	PAO1	MRSA	CRPA
amoxicillin	>0.5, S	512, R	>0.5, S	512, R
nitrofurantoin	8, S	N/A	32, R	N/A
ciprofloxacin	>0.5, S	>0.5, S	16, R	32, R
levofloxacin	>0.5, S	>0.5, S	4, R	64, R
cefazolin	>0.5, S	>1024, R	>0.5, S	>1024, R
ceftazidime	16, R	1, S	64, R	16, R
chloramphenicol	8, S	32, R	8, S	512, R
gentamicin	2, S	1, S	>0.5, S	>0.5, S
doxycycline	>0.5, S	N/A	>0.5, S	N/A
imipenem	>0.5, S	32, R	>0.5, S	256, R
meropenem	N/A	2, S	N/A	16, R
doripenem	N/A	2, S	N/A	16, R

a R = resistant, S = sensitive, N/A = not available.

The strains examined include *S. aureus* ATTC, *P. aeruginosa* PAO1, methicillin-resistant *S. aureus* (MRSA), and carbapenem-resistant *P. aeruginosa* (CRPA). While *S. aureus* ATTC demonstrates susceptibility to several antibiotics, including amoxicillin, nitrofurantoin, gentamicin, and chloramphenicol, MRSA shows resistance to most antibiotics tested.

PAO1 exhibits susceptibility to gentamicin and other carbapenems such as meropenem and doripenem. However, it is resistant to amoxicillin, imipenem, cefazolin, and chloramphenicol. In contrast, the carbapenem-resistant strains (CRPA) display resistance to a wide range of antibiotics, including ciprofloxacin, levofloxacin, cefazolin, ceftazidime, chloramphenicol, imipenem, meropenem, and doripenem. It is susceptible to only gentamicin among the antibiotics tested.

### SH-aPDT Effectively Inactivated Gram-Positive and Gram-Negative MDR Biofilms

**Figure 2:**
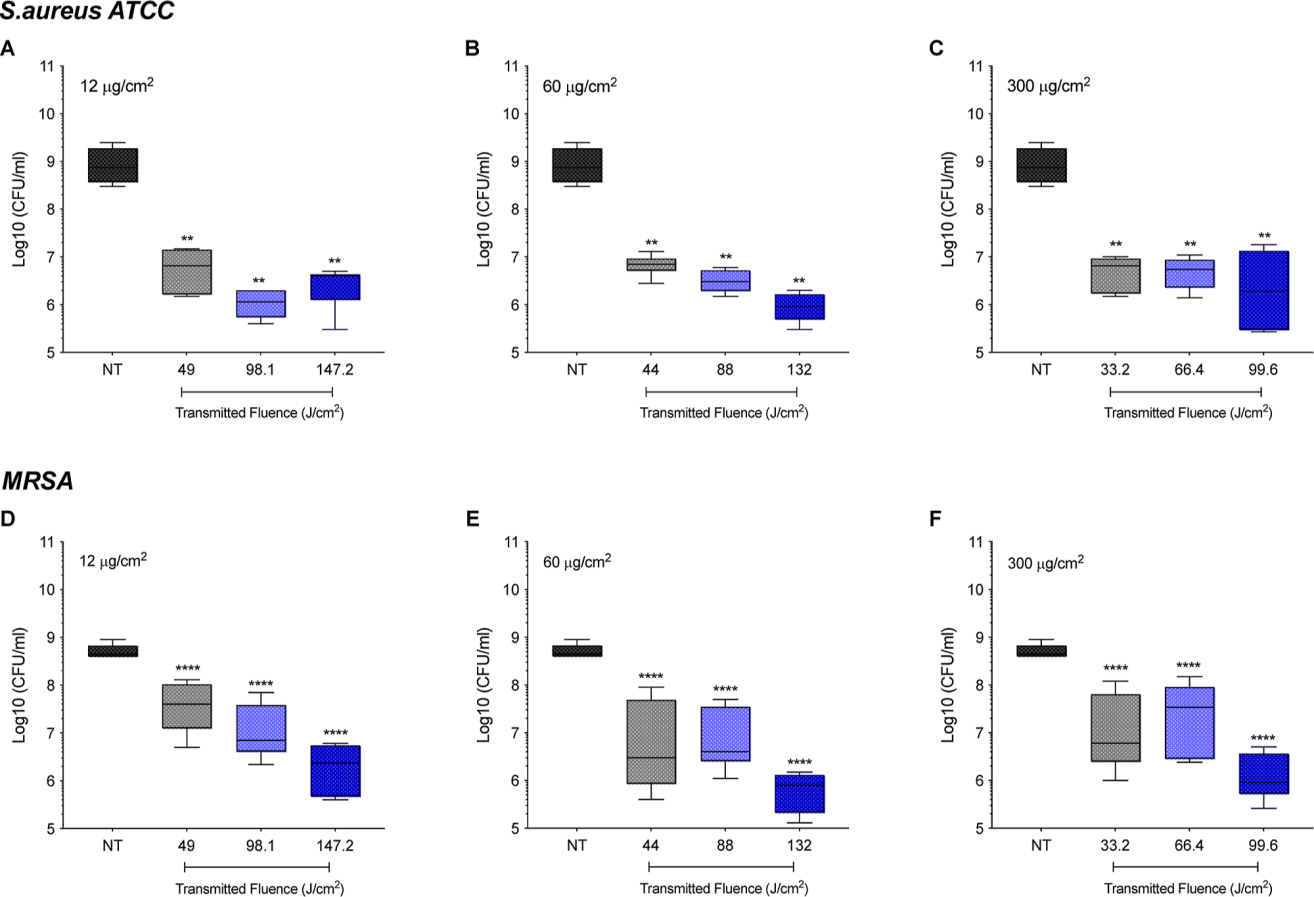
Efficacy of SH+PS+L+ surfaces coated with (A,D) 12, (B,E) 60, and (C,F) 300 μg/cm^2^ of VP against biofilms of *S. aureus* and MRSA at different fluences (J/cm^2^). Statistically significant differences: **p < 0.01 and ****p < 0.0001 between SH-aPDT and NT.

Our results show that SH-aPDT successfully inactivated biofilms of Gram-positive and Gram-negative MDR strains. All controls, including SH surfaces alone (SH+PS–L−) and coated with different loadings of PS and without light (SH+PS+L−), did not induce any significant effect on the bacterial specimens (Supporting Information, Figure S1). The efficacy of treatment was dependent on the VP loading and fluence, as shown in Figure 2. The lowest loading (12 μg/cm^2^) and TF 49 J/cm^2^ caused a 2.1 and 2.0 log_10_ reduction of *S. aureus* and MRSA, respectively, relative to NT (*p* < 0.01, *p* < 0.0001, respectively). The effect was more pronounced at the highest light dose (147.2 J/cm^2^), achieving nearly 2.5 and 2.3 log_10_ inactivation in both Gram-positive strains (ATCC *p* < 0.01, MRSA *p* < 0.0001) (Figure 2A,D). Remarkably, we observed that the VP loading of 60 μg/cm^2^ was more effective at the highest TF (132 J/cm^2^), leading to a 3 log reduction (ATCC *p* < 0.01, MRSA *p* < 0.0001) (Figure 2B,E). In contrast, no improvement was noticed under a higher PS loading (300 μg/cm^2^) (Figure 2C,F). MRSA killing showed a greater dependency on fluence for all PS loadings in comparison to *S. aureus*.

**Figure 3:**
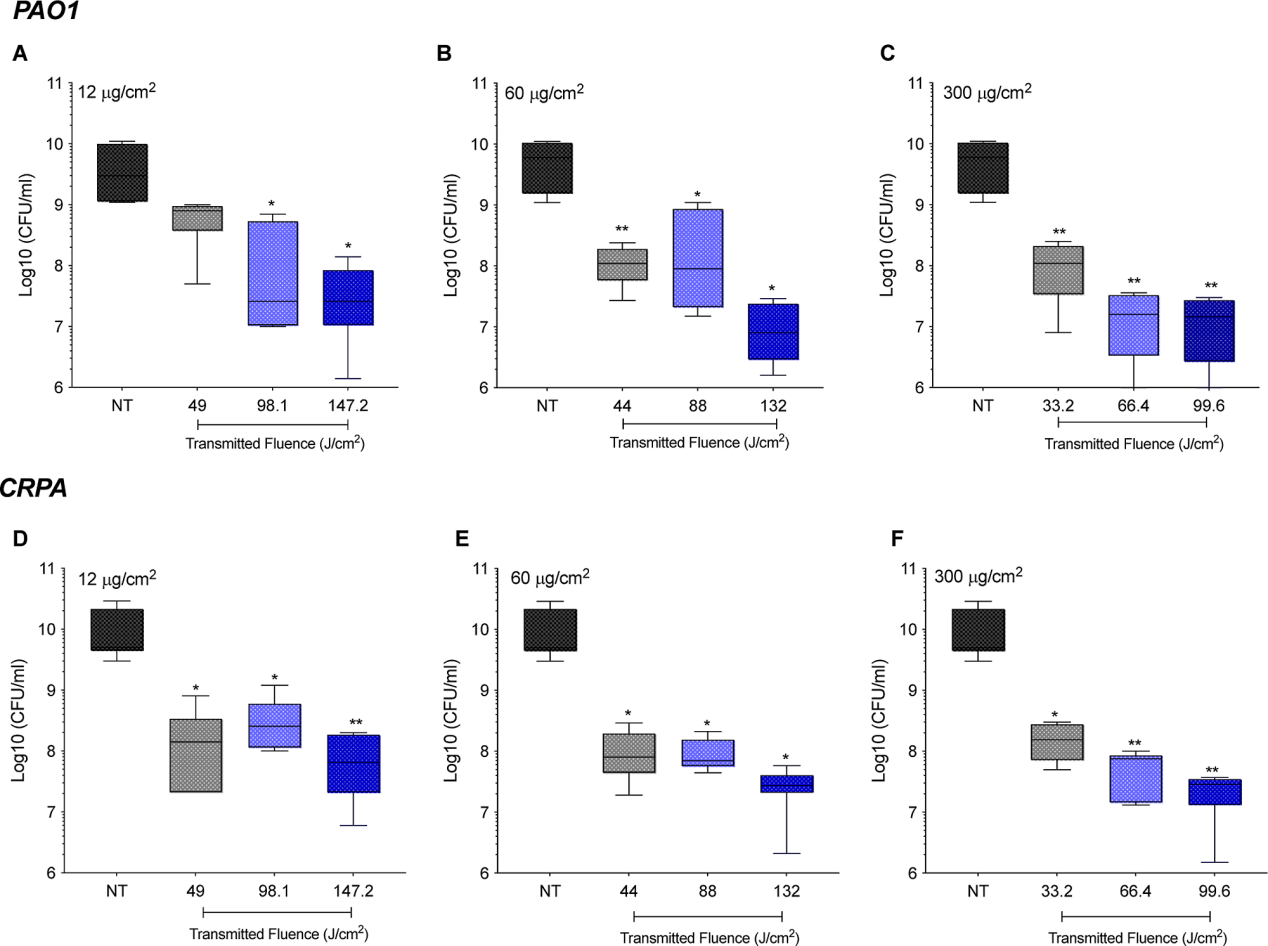
Efficacy of SH+PS+L+ surfaces coated with (A,D) 12, (B,E) 60, and (C,F) 300 μg/cm^2^ of VP against biofilms of *P. aeruginosa* PAO1 and CRPA at different fluences (J/cm^2^). Statistically significant differences: *p < 0.05 and **p < 0.01 between SH-aPDT and NT.

We also demonstrate that SH-aPDT effectively inactivated both strains of *P. aeruginosa* regardless of the PS loading (Figure 3), whereas no significant impact was observed with controls (SH–PS–L+, SH+PS–L+, SH+PS–L–, and SH+PS+L−) on either strain, as shown in Supporting Information, Figure S1.

For PAO1 at the lowest loading (12 μg/cm^2^, Figure 3A), no significant differences were observed between NT and biofilms exposed to 49 J/cm^2^. However, by increasing the light dose, biofilms were reduced by approximately 2 log_10_ when compared to NT or SH+PS–L+ (*p* < 0.05) (Figure 3A). SH-aPDT was more effective when PAO1 biofilms were exposed to SH surfaces coated with VP at higher loading levels. The increase in PS loading resulted in significant bacterial inactivation by 1.6 log_10_ at the lowest fluence (44 and 33.2 J/cm^2^) (*p* < 0.01), while at the highest light dose (132 and 99.6 J/cm^2^), more than 2.7 and 2.5 log_10_ reduction was achieved at loadings of 60 and 300 μg/cm^2^, respectively (*p* < 0.05 and *p* < 0.01, respectively) (Figure 3B,C).

SH-aPDT also showed high bacterial killing against the CRPA strain, which was significantly reduced by 1.7 log_10_ at the lowest transmitted light dose (49 J/cm^2^) and VP loading (12 μg/cm^2^) (*p* < 0.05) (Figure 3D). The killing rate was greatly enhanced by increasing the light dose and PS loading to 60 and 300 μg/cm^2^, thus showing 2.6 and 2.7 log_10_ reduction under a higher TF of 132 and 99.6 J/cm^2^, respectively (*p* < 0.05 and *p* < 0.01, respectively) (Figure 3E,F).

### SH-aPDT Reduced Metabolic Activity of Gram-Positive and Gram-Negative MDR Biofilms

**Figure 4:**
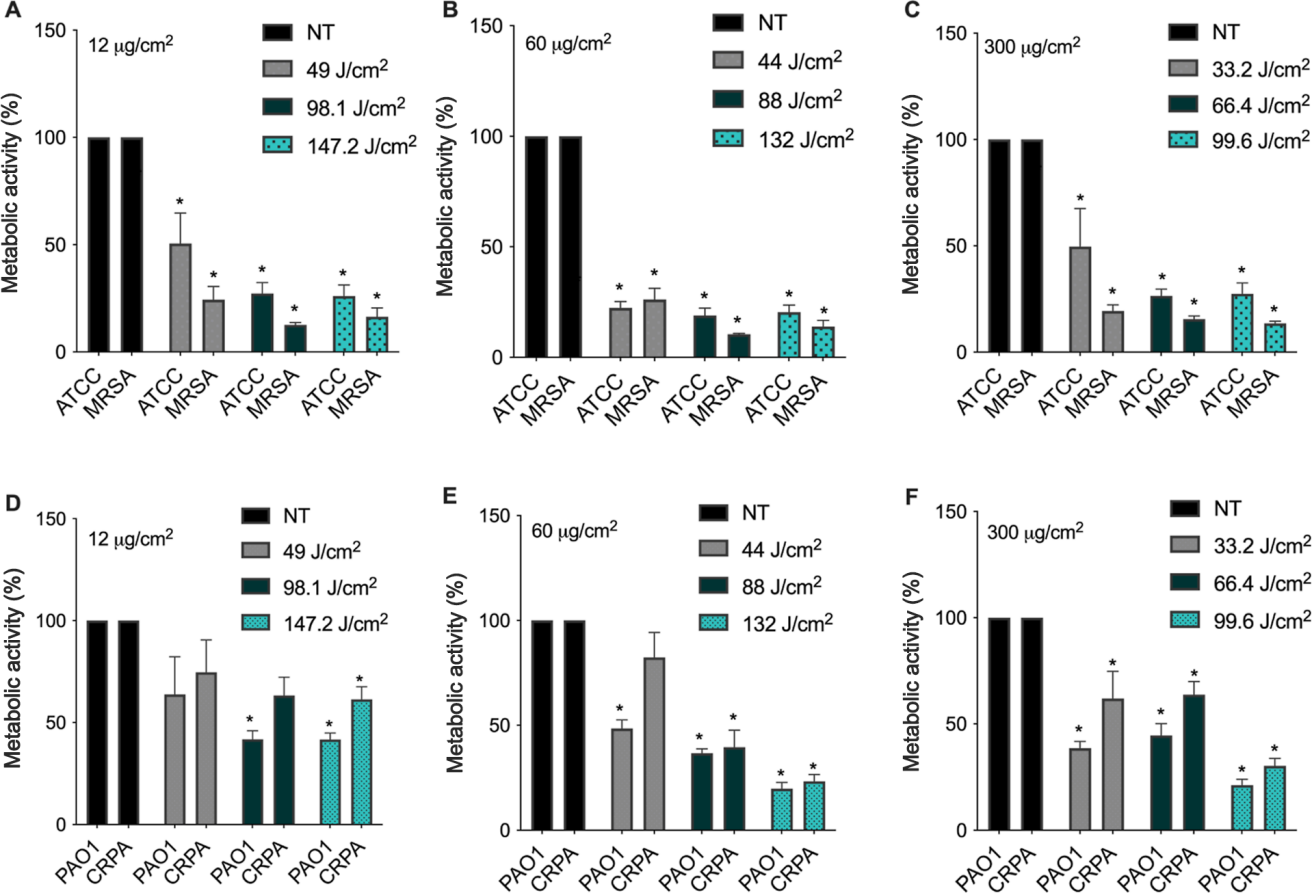
Metabolic activity after SH-aPDT killing of biofilms of drug-sensitive and drug-resistant *S. aureus* and *P. aeruginosa* at different TFs (J/cm^2^) with PDMS coated with (A,D) 12, (B,E) 60, and (C,F) 300 μg/cm^2^. * denotes statistically significant differences compared to the respective NT (*p* < 0.05).

SH-aPDT effectively reduced biofilm metabolism, as shown in Figure 4. At the lowest (12 μg/cm^2^) PS loading, significant decreases were observed for both Gram-positive strains (*S. aureus* and MRSA, Figure 4A), whereas the decreases were not significant for the Gram-negative strains (PAO1 and CRPA) (Figure 4D), which is consistent with the CFU results (Figures 2 and 3). Nevertheless, higher VP loadings resulted in a significant decrease in the measured metabolism for all strains. The best condition was for the loading of 60 μg/cm^2^, in which both *S. aureus* ATCC and MRSA showed a considerable decrease in their metabolism regardless of the light dose (Figure 4B). At 132 J/cm^2^, *S. aureus* ATCC activity was reduced by 81.7%, while MRSA showed 86% decrease (Figure 4B). Under the same conditions, the viability of PAO1 and CRPA was diminished by about 80.2 and 73.7% (Figure 4E). No additional improvement was noticed when the PS loading was increased to 300 μg/cm^2^ (Figure 4C,F). The other controls SH+PS–L–, SH+PS–L+, SH–PS–L–, and SH coated with different loading (SH+PS+12,60,300L−) did not promote any significant killing (Supporting Information, Figure S2).

### SH-aPDT Reduces Biofilm Thickness and Promotes Disruption in Their Structure

**Figure 5:**
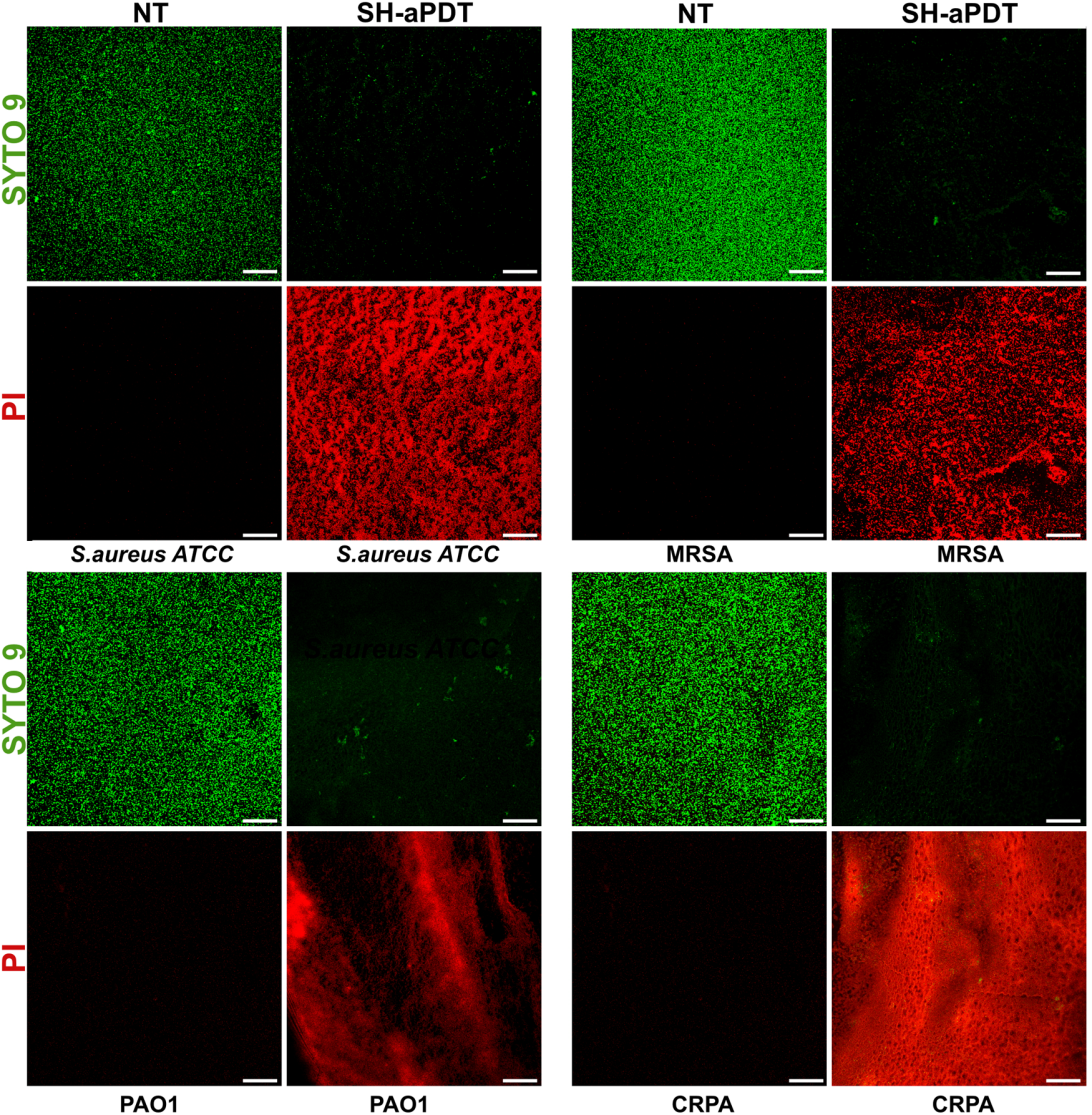
Confocal microscopy images of *S. aureus* ATCC, MRSA, PAO1, and CRPA biofilms stained with LIVE/DEAD after SH-aPDT 60 μg/cm^2^ of VP and a TF of 132 J/cm^2^ compared to NT. Scale bar = 30 μm.

Biofilm viability was also assessed via confocal microscopy. Bacterial specimens were stained with fluorescent dyes SYTO 9 and PI. While SYTO 9 stains live bacteria and emits green fluorescence, and dead bacteria are marked with PI, thus emitting red fluorescence. Images are consistent with the metabolic activity and CFU counting described above, in which most of the biofilms were dead (indicated by red fluorescence) under the highest fluence and 60 μg/cm^2^ PS. In addition, SH-aPDT induced disruption in biofilms, leading to irregularities, increased roughness, and greater porosity of the surface compared to untreated control (Figure 5).

**Figure 6:**
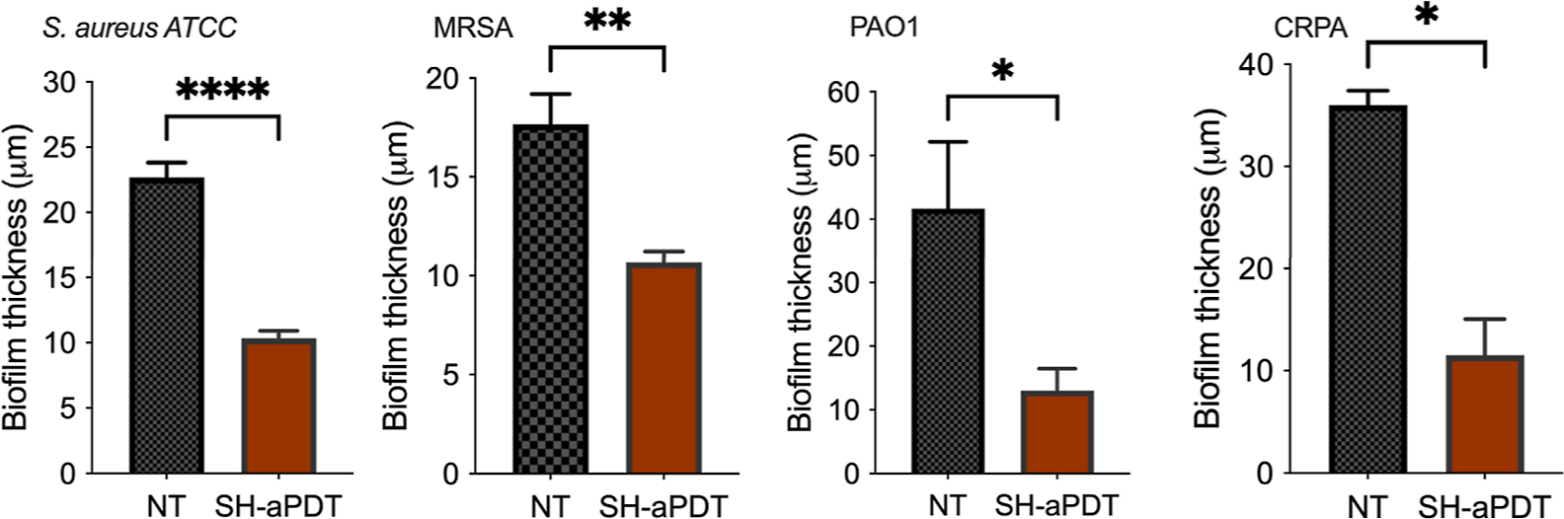
Biofilm thickness of drug-sensitive and drug-resistant biofilms of Gram-positive and Gram-negative bacteria after SH-aPDT treatment using 60 μg/cm^2^ of VP and a TF of 132 J/cm^2^. Statistically significant differences: *p < 0.05, **p < 0.01, and ****p < 0.0001 between SH-aPDT and NT.

Biofilm thickness was measured by capturing 3D (z-stack) images and then examining them to determine the thickness. Figure 6 shows that biofilm thickness was substantially reduced after SH-aPDT, resulting in 54.4 and 39.6% reduction for *S. aureus* ATCC and MRSA, respectively. Surprisingly, the impact was more pronounced in Gram-negative strains, with a 68 and 68.8% reduction in biofilm thickness of PAO1 and CRPA, respectively.

### SH-aPDT Significantly Decreases Biofilm Biomass

**Figure 7:**
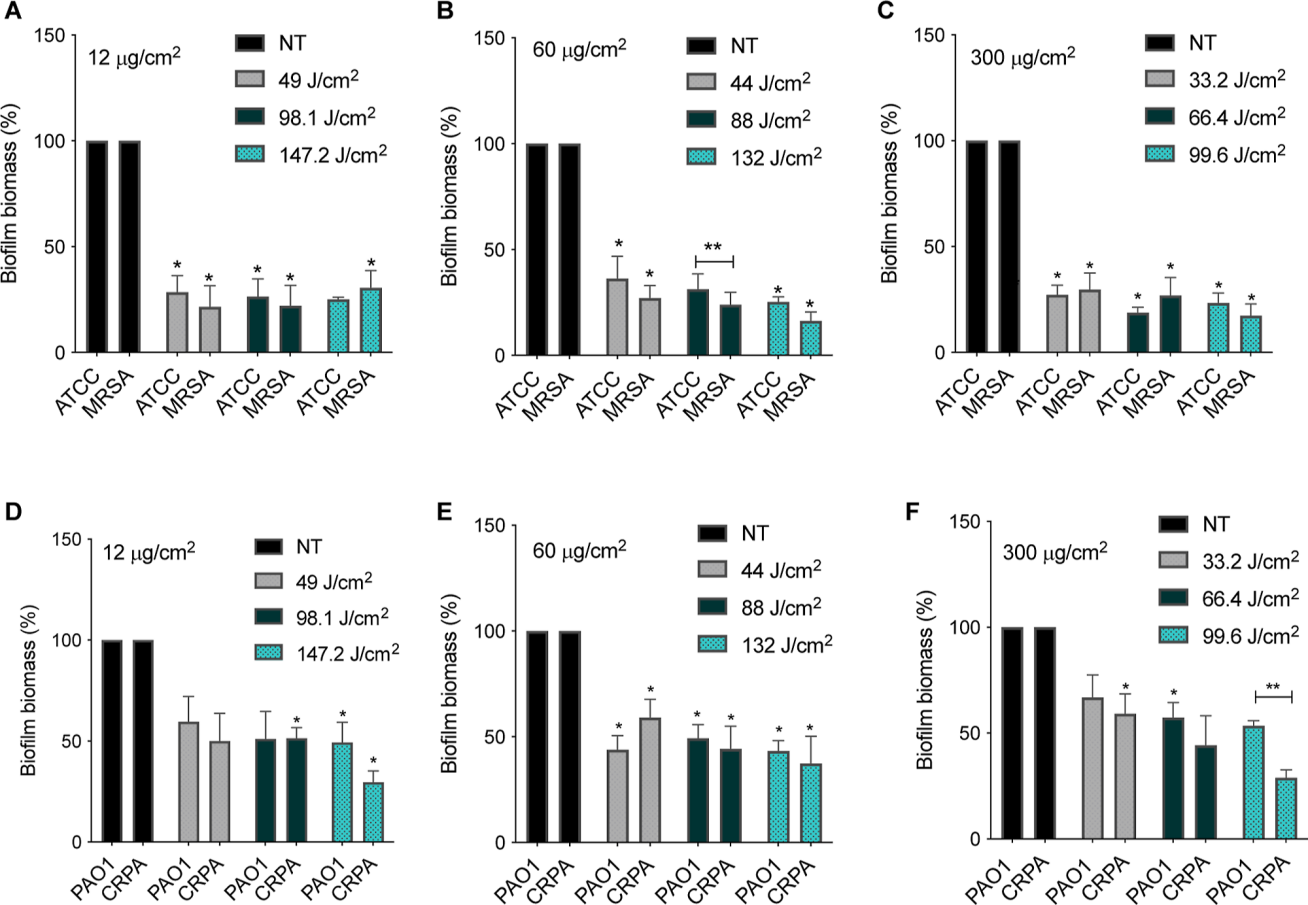
Efficacy of SH PDMS surfaces coated with (A,D) 12, (B,E) 60, and (C,F) 300 μg/cm^2^ of VP on biofilms biomass of *S. aureus* ATCC, MRSA, *P. aeruginosa* PAO1, and CRPA at different TFs (J/cm^2^). * denotes statistically significant differences compared to the respective control. ** denotes statistically significant differences between different strains (*p* < 0.05).

We observed that SH-aPDT promoted a significant decrease in the biomass of both drug-sensitive and drug-resistant biofilms, as shown in Figure 7. Controls (SH+PS–L–, SH+PS–L+, SH–PS–L–, and SH) coated with different VP concentrations (12, 60, and 300 μg/cm^2^) did not result in any significant biofilm mass (Supporting Information, Figure S3). We found that *S. aureus* and MRSA biomass decreased by >64% at all PS loading levels and all fluence values. Gram-negative *P. aeruginosa* strains followed the same trend, but the magnitudes of the biomass decrease were smaller <41%. At 300 μg/cm^2^ and 132 J/cm^2^, a 56.7% decrease in biomass was observed in the drug-sensitive strain, while for the drug-resistant line, a 53.1% decrease was achieved.

### SH-aPDT Promotes Disruption of the EPS Matrix of Biofilms

**Figure 8:**
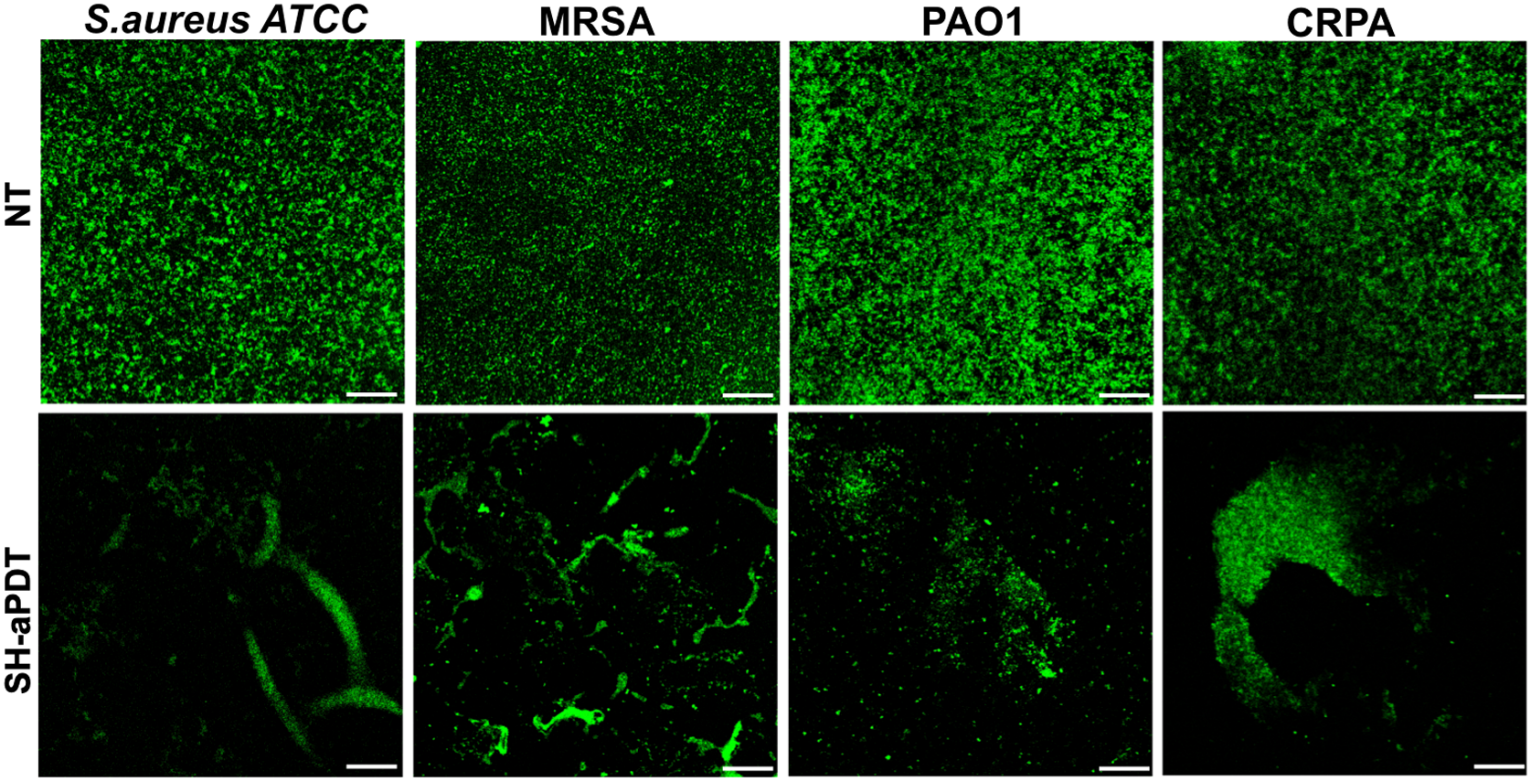
Confocal microscopy images of drug-sensitive and drug-resistant biofilms stained with FilmTracer SYPRO RUBY biofilm matrix stain after SH-aPDT 60 μg/cm^2^ of VP and 132 J/cm^2^. Scale bar = 30 μm.

SH-aPDT also had a pronounced impact on the structure of the EPS matrix of all four strains (Figure 8). Exposure of bacterial specimens to a fluence of 132 J/cm^2^ and 60 μg/cm^2^ of VP resulted in significant damage to the EPS. Untreated controls exhibited mature biofilms with preserved architecture, while treated ones displayed loss of their structure and smaller aggregates. Microscopy findings are consistent with reductions in the biofilm thickness (Figure 6) and biomass (Figure 7).

EPS plays an important role in physical stability, resistance to antimicrobials, mechanical removal, and host immunity, thus having significant implications for wound healing. Therefore, disrupting the biofilm structure would make the pathogens more susceptible to drugs, facilitate mechanical removal, and, thus, enhance treatment efficacy.

### Singlet Oxygen Quenching by Sodium Azide

**Figure 9:**
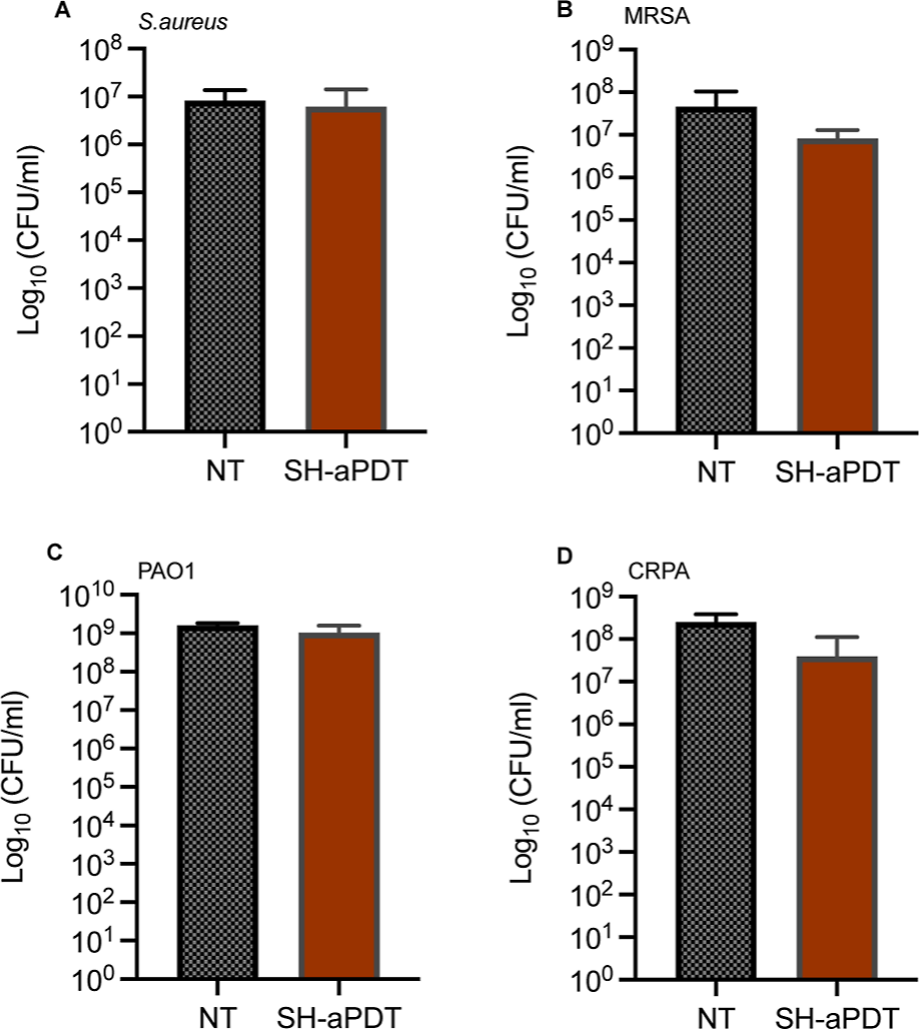
Activity of SH PDMS surfaces coated with 60 μg/cm^2^ of VP against biofilms of drug-sensitive and drug-resistant bacteria at a TF of 132 J/cm^2^ in the presence of the ^1^O_2_ quencher sodium azide (2 mM) (A) *S. aureus*, (B) MRSA, (C) PA01, and (D) CRPA.

To evaluate whether the killing effect originated from airborne ^1^O_2_ delivery, we added sodium azide (a ^1^O_2_ quencher) to the biofilms before illumination. Consequently, we observed (Figure 9) that no killing occurred compared to the controls, which is consistent with the quenching of ^1^O_2_ and the prevention of biofilm death. Although we observed a slight decrease in the average bacterial load (≤0.8 log_10_) for MRSA and CRPA, this reduction was not statistically significant.

### HPF Detection of Downstream Oxygen Radicals

**Figure 10:**
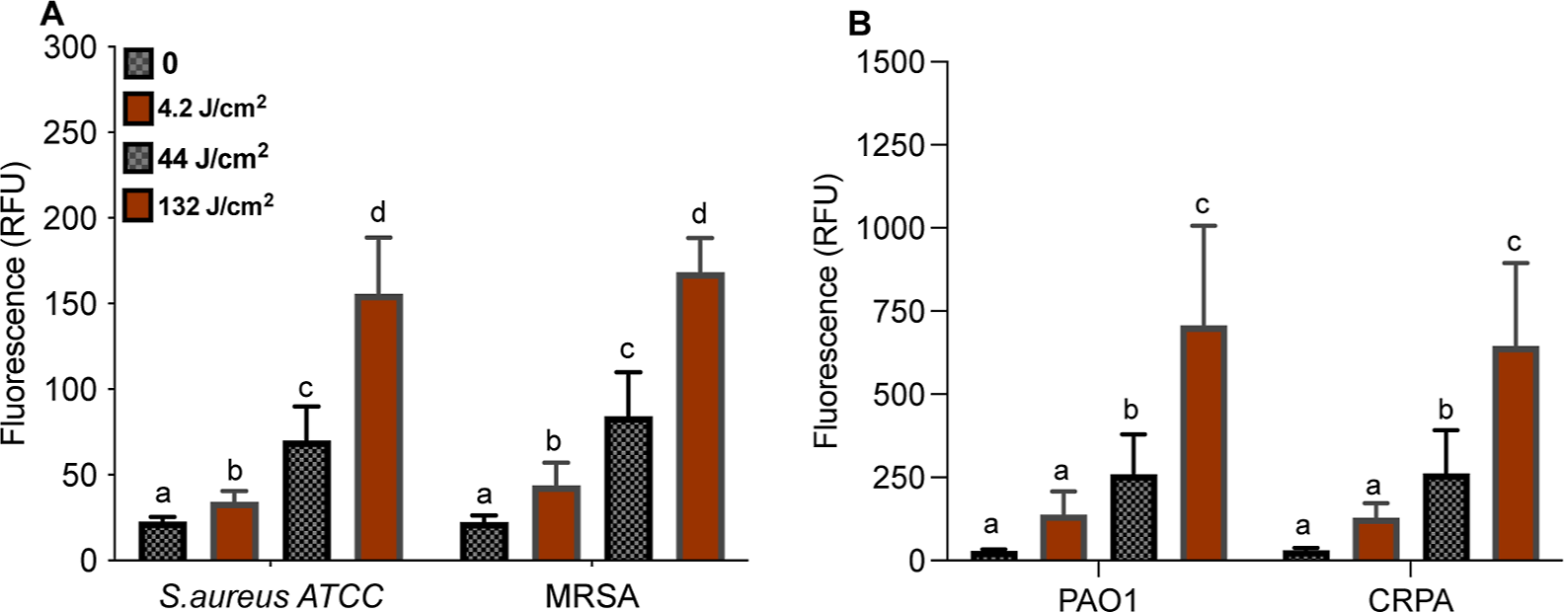
HPF fluorescence on biofilms resulting from SH-aPDT (60 μg/cm^2^ of VP) and different light doses for (A) *S. aureus* and (B) *P. aeruginosa*. Mean values not sharing any letters indicate a significant statistical difference.

Figure 10 shows the HPF fluorescence results from biofilms treated with SH-aPDT. At 132 J/cm^2^, there was an 8.2-fold and 7.4-fold increase in these radicals after SH-aPDT treatment of *S. aureus* ATCC and MRSA biofilms (Figure 10). The effect was more pronounced in Gram-negative strains, in which the levels of radicals were nearly 26-fold higher compared with the untreated control group. The significant increase suggests that ^1^O_2_ first reacts with lipids or some amino acid sites/proteins within the biofilm to form bioperoxides that subsequently decay to form oxygen radicals detected by HPF.

## Discussion

In the present work, we demonstrate that SH-aPDT is a highly effective antimicrobial method for killing MDR biofilms of *S. aureus* and *P. aeruginosa*, which are among the most common drug-resistant pathogens that cause high mortality rates in patients. MRSA and CRPA are considered significant public health threats and are included in the high and critical priority list of the World Health Organization (WHO) for drug discovery and research, respectively.^24^ This highlights the urgent need for developing alternative strategies to combat these antibiotic-resistant pathogens.

While aPDT has demonstrated promising results in multiple MDR strains, the efficacy of aPDT is dependent upon the PS’s uptake by bacteria, which could lead to the development of resistance. Our SH-aPDT system eliminates the need for direct PS contact with the tissue, where we established its efficacy in a dose-dependent manner, with a TF of 132 J/cm^2^ at a VP loading of 60 μg/cm^2^, resulting in a >3 log reduction.

SH-aPDT also led to a substantial reduction in biofilm thickness and a significant decrease in metabolic activity against all of the tested strains. Biofilm-associated bacteria are difficult to treat and inherently more tolerant to antimicrobials, including antibiotics.^4,25^

Reducing biofilm thickness is one of the primary goals of treatment strategies because thicker biofilms act as a physical barrier, limiting drug penetration and thus contributing to increased resistance. Thicker biofilms can provide a protective environment due to the EPS matrix that prevents antimicrobials and the host immune cells from effectively targeting and eradicating bacteria, thereby resulting in greater survival and resistance to therapy.^4,25^

The efficacy of antimicrobial treatments depends on the biofilm’s age, where a number of studies indicate that 48 h biofilm is considered to be mature for the strains *S. aureus* and *P. aeruginosa*.^26−29^ Not only does SH-aPDT cause a significant reduction in biofilm thickness but it also results in a disruption of the biofilm structure and a significant reduction in the biomass regardless of the PS or light dose applied. The efficacy of antimicrobial treatments depends on the biofilm’s age, where a number of studies indicate that 48 h biofilm is considered to be mature for the strains *S. aureus* and *P. aeruginosa*. This highlights the effectiveness of SH-aPDT not only in inactivating bacteria but also in disrupting the structure of the biofilms, including the EPS matrix. The outcome was consistent with confocal microscopy, in which obvious EPS damage was observed following treatment. This means that SH-aPDT should facilitate the detachment of biofilms from the wound surfaces. Furthermore, the disruption of EPS provides opportunities for the penetration of antimicrobials, thereby enhancing the efficacy of adjuvant treatments.

SH surfaces are reported to emit airborne ^1^O_2_.^18,30^ A mechanistic assessment of SH-aPDT to account for the above results is the initial contact between airborne ^1^O_2_ and the biofilm. In a previous report,^18^ we measured the 1270 nm luminescence intensity within a SH system, confirming the presence of airborne ^1^O_2_. We found that this airborne ^1^O_2_ could be transported from the PS-coated SH surface and chemically trapped in an aqueous droplet positioned up to 0.6 mm away, which is consistent with airborne ^1^O_2_ with a lifetime of ∼0.7 ms instead of much shorter-lived oxygen radicals.

Airborne ^1^O_2_ can react with multiple sites within the EPS matrix. The significant reduction in phototoxicity in the presence of sodium azide supports ^1^O_2_ as the main species responsible for bacterial killing (Figure 9), which is consistent with prior VP studies.^31,32^

Sodium azide deactivates ^1^O_2_ by converting it to ground-state ^3^O_2_ by physical quenching.^33^ The results also suggest the formation of bioperoxides, as well as their subsequent decomposition. The HPF studies point to bioperoxide decomposition after ^1^O_2_ exposure with downstream detection of oxygen radicals that arise from peroxidized bacterial membranes within the EPS. Such peroxides can decompose over time by radical chain and other processes.^16,34−41^ Thus, we suggest that bioperoxides diffusing through the biofilms and subsequently forming oxygen radicals can contribute to bacterial killing.

The airborne ^1^O_2_ produced from the SH surface has a limited diffusion distance of approximately 1 mm in air but is about 10^4^ times shorter in an aqueous solution (100–200 nm)^9,19^ While SH-aPDT offers the advantage of introducing the key species thought to underlie PDT, it does so without the PS directly contacting the tissue. Uptake of the PS by the bacteria is avoided; thus, this aPDT limitation is overcome.^17,18^

## Conclusions

SH-aPDT is emerging as an attractive strategy to overcome some of the limitations of aPDT for the treatment of drug-resistant infectious diseases. The innovative SH-aPDT system offers a promising approach to treat wounds infected by medically relevant MDR bacteria by delivering ^1^O_2_ directly into the target while minimizing contact with surrounding tissues. Future studies will be aimed at assessing the potential of SH-aPDT for in vivo (animal model) applications. The SH membrane is made of medical-grade PDMS, thereby ensuring safety and facilitating acceptance. The present work is similar to our previous report on the use of VP for the treatment of the Wistar rat periodontal disease model.^17^ This PS is a component of an FDA-approved treatment of age-related macular degeneration.^42^ Notably, the quantity of VP applied to the SH membrane is <0.1% of the FDA-approved dose for intravenous use for age-related macular degeneration,^43^ indicating a safe and low-risk treatment method for patients.

## Supporting Information Available

Details on SH and light controls on colony-forming units, metabolic activity of Gram-positive and Gram-negative drug-resistant biofilms, and biofilm biomass reduction (PDF)
