# Supplementary material for: Superhydrophobic Dressing for Singlet Oxygen Delivery in Antimicrobial Photodynamic Therapy against Multidrug-Resistant Bacterial Biofilms
Source: ACS Appl Bio Mater. 2024 Aug 21;7(9):6175–85. doi: 10.1021/acsabm.4c00733 (PMC11409211; doi:10.1021/acsabm.4c00733)
Supplement: Supplementary file 1 — mt4c00733_si_001.pdf [file mt4c00733_si_001.pdf]

Supporting information

Superhydrophobic Dressing for Singlet Oxygen Delivery in Antimicrobial Photodynamic Therapy against Multidrug-resistant Bacterial Biofilms

Fernanda V. Cabral[1], QianFeng Xu[2], Alexander Greer[2,3,4]*, Alan M. Lyons[2,3,5]* and Tayyaba Hasan[1,6]*

1 - Wellman Center for Photomedicine, Massachusetts General Hospital and Harvard Medical School, 40 Blossom Street, Boston, Massachusetts 02114, United States.

2 - SingletO2 Therapeutics LLC, VentureLink, Room 524B, 211 Warren Street, Newark, New Jersey 07103, United States.

3 - Ph.D. Program in Chemistry, The Graduate Center of the City University of New York, 365 Fifth Avenue, New York, New York 10016, United States.

4 - Department of Chemistry, Brooklyn College, City University of New York, Brooklyn, New York 11210, United States.

5 - Department of Chemistry, College of Staten Island, City University of New York, Staten Island, New York 10314, United States.

6 - Division of Health Sciences and Technology, Harvard University and Massachusetts Institute of Technology, Cambridge, Massachusetts 02139, United States.

E-mail addresses: greer@singletO2.com (A. Greer), alan@singletO2.com (A. M. Lyons), thasan@mgh.harvard.edu (T. Hasan)

Colony-forming units remain unaffected by SH and light controls

We evaluated the impact of various control conditions on four bacterial strains: non-treated (NT), light exposure only (SH-PS-L+), SH coated with 12 µg/cm², 60 µg/cm², and 300 µg/cm² of verteporfin (SH+PS+L+), and PDMS only (SH+PS+L-). For all strains, the bacterial load remains consistently high across all conditions, with slight variations. This indicates that these conditions do not significantly affect bacterial viability.

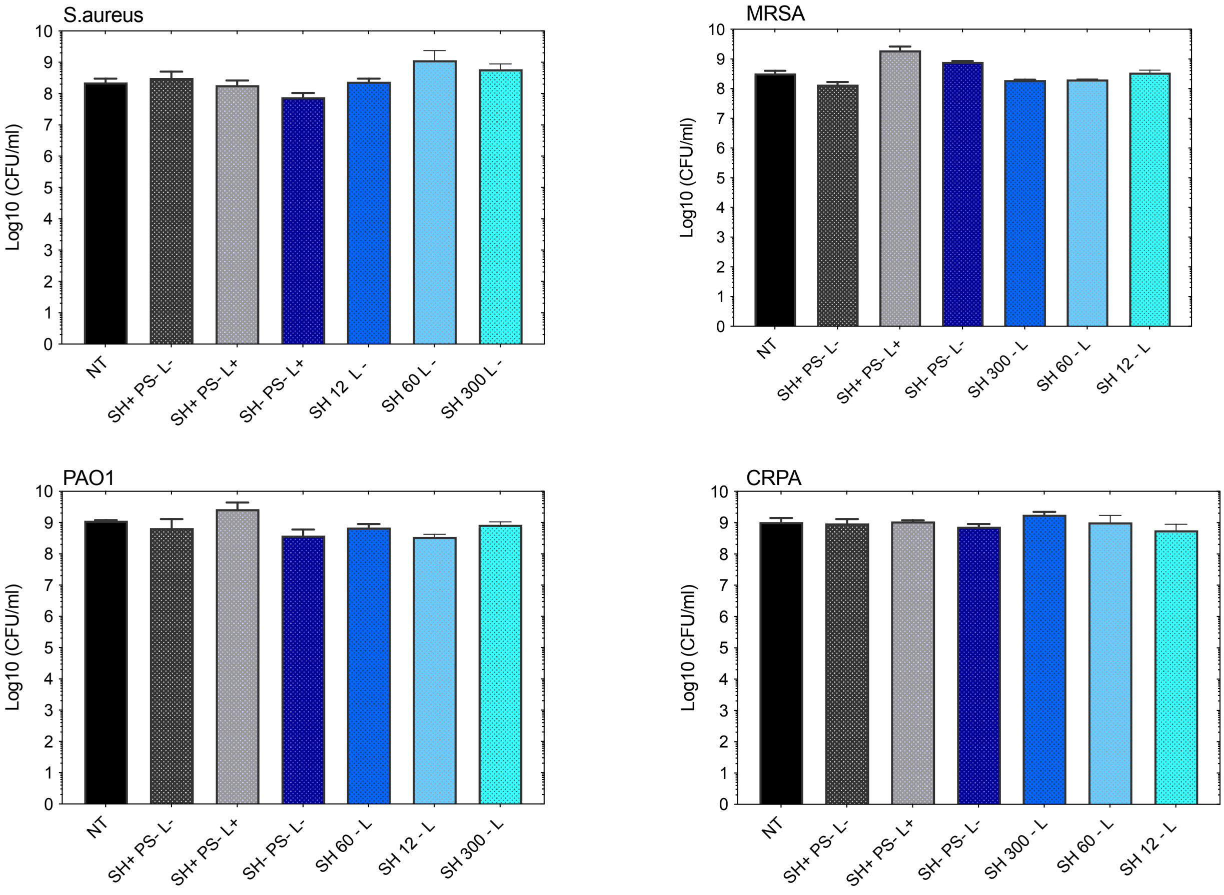

Figure S1. Bacterial load (log_{10} CFU/ml) of biofilms of *S.aureus* and MRSA, PAO1 and CRPA under different control conditions, including non-treated (NT), SH + PS-L- (SH with PS, no light exposure), SH + PS-L+ (SH with PS, light exposure), SH 12 L- (SH at 12 µg/cm² without light), SH 60 L- (SH at 60 µg/cm² without light), and SH 300 L- (SH at 300 µg/cm² without light).

SH and light controls did not reduce the metabolic activity of gram-positive and gram-negative drug-resistant biofilms

This figure presents bar graphs showing the metabolic activity assessed by MTT of four different strains under various control conditions. Each graph represents a different bacterial strain: S. aureus, MRSA, PAO1, and CRPA. The x-axis labels denote different control conditions, while the y-axis represents the percentage of metabolic activity. The consistent high metabolic activity across all conditions indicates that the tested control treatments (SH, PS, light exposure, and different concentrations of SH) do not significantly impact the viability of S. aureus, MRSA, PAO1, and CRPA.

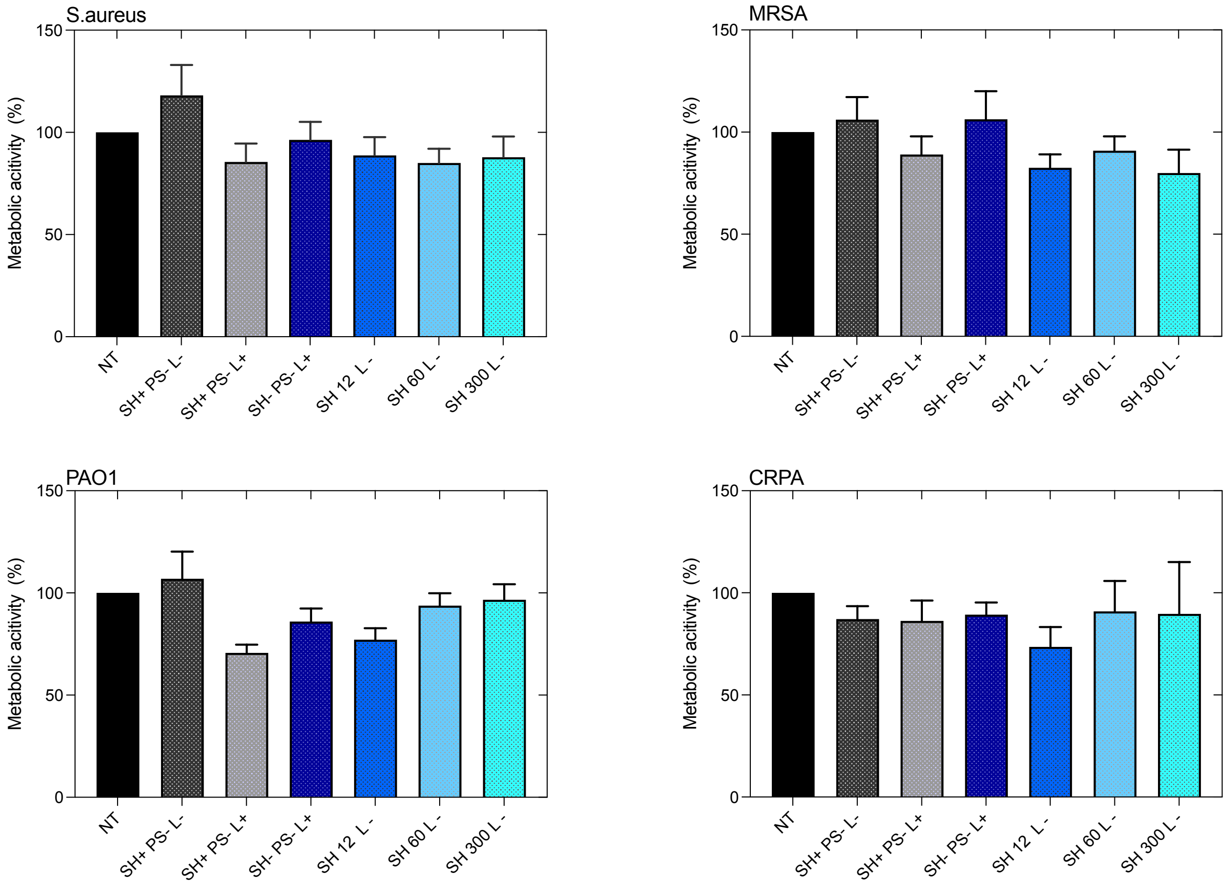

Figure S2. Metabolic activity of biofilms of *S. aureus* and MRSA, PAO1 and CRPA under different control conditions, including non-treated (NT), SH + PS-L- (SH with PS, no light exposure), SH + PS-L+ (SH with PS, light exposure), SH 12 L- (SH at 12 µg/cm² without light), SH 60 L- (SH at 60 µg/cm² without light), and SH 300 L- (SH at 300 µg/cm² without light).

SH and light controls did not reduce biofilm biomass

We also evaluated biofilm biomass under different control conditions. These findings confirm that the control treatments, including SH, PS, light exposure, and different loadings of SH, do not significantly influence the biofilm biomass of S. aureus, MRSA, PAO1, and CRPA.

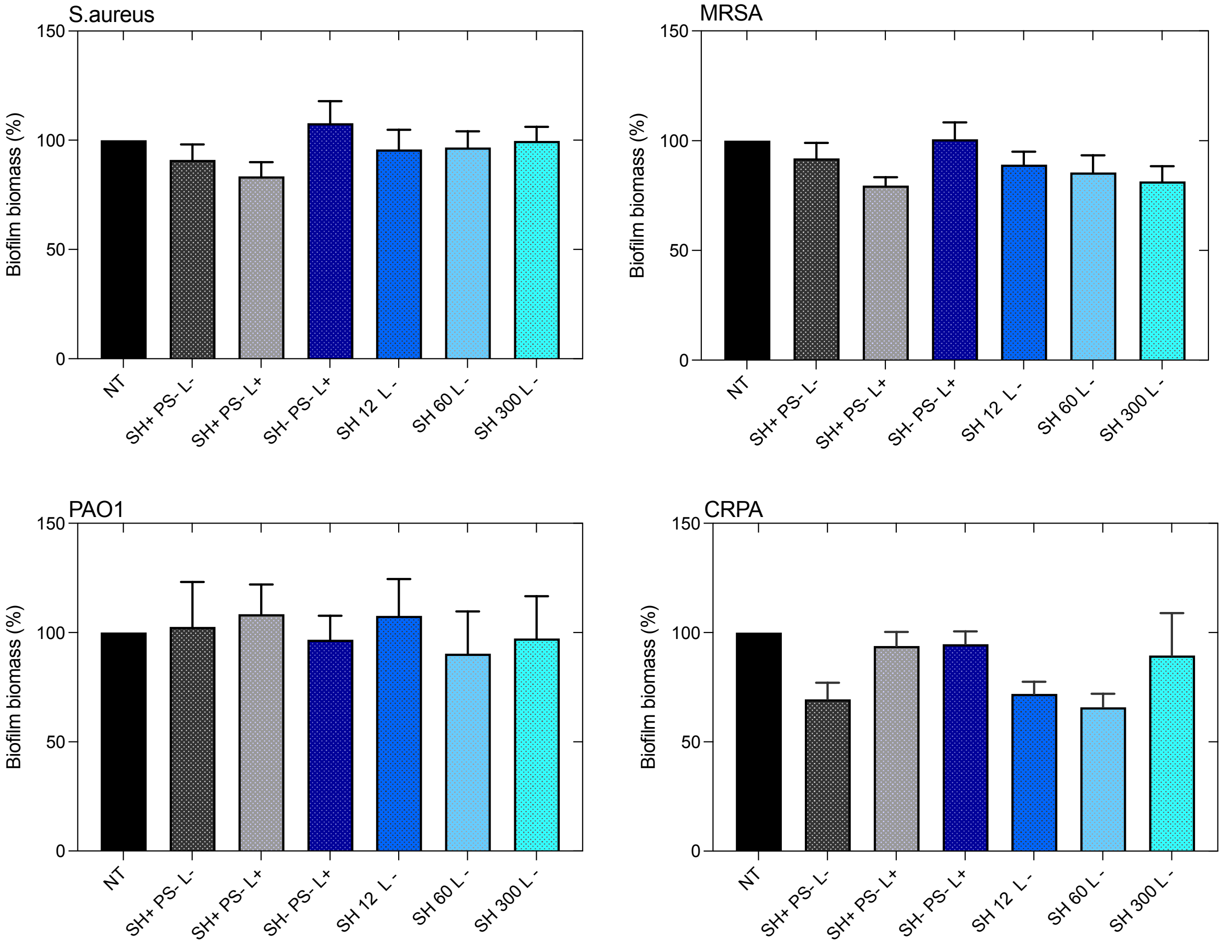

Figure S3. Biofilms biomass of *S. aureus* and MRSA, PAO1 and CRPA under different control conditions, including non-treated (NT), SH + PS-L- (SH with PS, no light exposure), SH + PS-L+ (SH with PS, light exposure), SH 12 L- (SH at 12 μg/cm² without light), SH 60 L- (SH at 60 μg/cm² without light), and SH 300 L- (SH at 300 μg/cm² without light).
